# Fault Detection of Wind Turbine Electric Pitch System Based on IGWO-ERF

**DOI:** 10.3390/s21186215

**Published:** 2021-09-16

**Authors:** Mingzhu Tang, Jiabiao Yi, Huawei Wu, Zimin Wang

**Affiliations:** 1 School of Energy and Power Engineering, Changsha University of Science & Technology, Changsha 410114, China; tmz@csust.edu.cn (M.T.); 20106011037@csust.edu.cn (J.Y.); 2 Hubei Key Laboratory of Power System Design and Test for Electrical Vehicle, Hubei University of Arts and Science, Xiangyang 441053, China; 3 School of Computer Science and Information Security, Guilin University of Electronic Technology, Guilin 541004, China; worthyman@guet.edu.cn

**Keywords:** wind turbine generator set, electric pitch system, extreme random forest, grey wolf optimization, fault detection

## Abstract

It is difficult to optimize the fault model parameters when Extreme Random Forest is used to detect the electric pitch system fault model of the double-fed wind turbine generator set. Therefore, Extreme Random Forest which was optimized by improved grey wolf algorithm (IGWO-ERF) was proposed to solve the problems mentioned above. First, IGWO-ERF imports the Cosine model to nonlinearize the linearly changing convergence factor α to balance the global exploration and local exploitation capabilities of the algorithm. Then, in the later stage of the algorithm iteration, α wolf generates its mirror wolf based on the lens imaging learning strategy to increase the diversity of the population and prevent local optimum of the population. The electric pitch system fault detection method of the wind turbine generator set sets the generator power of the variable pitch system as the main state parameter. First, it uses the Pearson correlation coefficient method to eliminate the features with low correlation with the electric pitch system generator power. Then, the remaining features are ranked by the importance of the RF features. Finally, the top N features are selected to construct the electric pitch system fault data set. The data set is divided into a training set and a test set. The training set is used to train the proposed fault detection model, and the test set is used for testing. Compared with other parameter optimization algorithms, the proposed method has lower FNR and FPR in the electric pitch system fault detection of the wind turbine generator set.

## 1. Introduction

As a renewable energy source, wind energy has many advantages, such as being pollution-free and renewable. It also has a wide distribution and large reserves, which lead to broad application prospects [1]. Because of poor working conditions, it is easy to cause damage to the components of the fan. If there is an accident, such as a shutdown caused by a fault, it will not only affect the normal operation of the wind turbine generator set, but also cost a lot in maintenance [2,3]. Therefore, it is of great significance to accurately detect the fault location of the wind turbine generator set.

As a subsystem of a large wind turbine generator set, the electric pitch system can maintain the rated power output and protect the system by controlling the adjustment of the pitch angle. Since the electric pitch system has a high failure rate, which can result in a long downtime, the fault detection becomes particularly important. Currently, the wind turbine fault detection (FD) methods can be divided into model-based fault detection and supervisory control and data acquisition (SCADA). The model-based fault detection method focuses on the combination of the internal models of the wind turbine, while the data-based fault detection method processes the original data, and obtains the correlation analysis of the processed data by different methods. Castellani, F. [4] established the relationship between wind turbine power, voltage, and current based on principal component analysis and support vector regression. The results show that the occurrence of fault can be individualized as the change of residual behavior between model estimation and measurement. Zhao, Y.Y. [5] designed an integrated prediction and diagnosis method, which can predict the service life of wind turbines through the SCADA system and diagnose the state of wind turbines when faults occur. Li, M. [6] used multiple Small-World Neural Networks (SWNNs) and the weighted integration method to train and classify the data collected by the system. The experimental results showed that the effective accuracy rate of this integration strategy can exceed 93.8% in a short time. Shi Yiran [7] proposed a pitch angle control system for the variable-speed wind turbine generator set based on an adaptive neural network with FD and fault tolerance. As for the failure of the pitch actuator of wind turbines, Wang [8] developed a multi-scale spatio-temporal convolution deep belief network to perform feature learning and classification tasks in order to address the difficulty of the SCADA system in fault detection due to multivariate time series with spatio-temporal correlation characteristics. In view of the faults of the electric pitch system, the above method integrates multiple learning strategies to analyze and detect a specific feature, but ignores the relationship between multiple sub-components in the electric pitch system, which has certain limitations.

The FD methods based on machine learning include the Artificial Neural Network [9,10], Random Forest [11,12,13], LightGBM [14], Deep Learning [15,16], and the Large Margin Distribution Machine [17,18]. J. H. Pan [9] designed a data-driven method based on a deep convolutional neural network (DCNN) for the gain error, position deviation, and the faults of sensors and actuators. This method can not only reduce the model training time, but also achieve a high fault recognition accuracy. To solve the problem of low efficiency and accuracy of traditional improvement algorithms during the processing of the large data of wind turbines, Tang, M.Z. [14] proposed an adaptive LightGBM FD model of the gearbox, whose results showed that the gearbox FD method based on the adaptive LightGBM had a low FPR and a low FNR. Considering the shortcomings of traditional fault diagnosis methods, Tang, S.N. [16] summarized the rotating machinery—such as bearing, gear/gearbox, and pumps FD methods based on deep learning—and carried out its prospect forecast. Because the FD method based on machine learning can analyze and process fuzzy faults without establishing an accurate model, it can better deal with the uncertainty and suddenness of random faults in the wind turbine generator set.

Random Forest is often used for classification and regression tasks in FD models. L.J. Wan [19] proposed a high-efficiency rolling bearing FD method based on Spark and improved random forest, which can increase the detection speed and obtain a higher accuracy. Ma, S.L. [20] developed an effective identification system based on wavelet packet technology and random forest by observing that high voltage circuit breakers are prone to mechanical faults in long-term operation. Ma, Suliang [21] focused on the problem that the vibration signal of a high voltage circuit breaker is easy to ignore, and subsequently proposed a hybrid feature transformation method to optimize the fault diagnosis performance of HVCB. Firstly, a random forest binary code is designed. Secondly, the feature depth is compressed by a laminated self-coding neural network, and is finally substituted into the data set for experiments. Qin, S.Y. [22] focused on the input/output and feature selection of the SCADA system, based on the general CMDF model. The random forest algorithm is used to find the best input feature, wavelet analysis is used for noise reduction, and an RLS filter is used to reduce the False Alarm Rate. Yan, Xiangwu [23] designed a fault diagnosis method combining multi-layer neural network and random forest based on the SCADA system. Wang, X.D. [24] focused on the difficult and time-consuming problem of the DOWF short-distance transmission line location, proposing a two terminal fault location method combining ST and RF. In order to do so, the fault features are first extracted from the collected wind turbine current signal through ST transform, and then the data are processed and transformed into the Random Forest model for diagnosis. The results show that this location method has a high fault identification rate. Mansouri, M. [25] focused on the shortcomings of the traditional random forest algorithm, and proposed a new random forest algorithm based on simplified Gaussian regression for the detection and diagnosis of the wind energy conversion system. Z.H. Xie [26] not only used RF for feature selection, but also proposed an improved cuckoo search (CS) algorithm to optimize the neural network parameters to avoid local optimum, which can significantly improve the accuracy of FD. To solve the problem of unbalanced data types in the wind turbine generator set, M.Z. Tang [10] proposed a cost-sensitive extreme random tree algorithm. Because of the randomness of RF, the actual operation of the model cannot be accurately known, and it is difficult to find a reasonable explanation for the assignment of different parameters. Therefore, the grey wolf optimization algorithm, which has a strong optimization ability, is combined with extreme random forest to optimize the FD model.

The grey wolf optimization is a swarm intelligence optimization algorithm proposed by Mirjalili in 2014, which has the advantages of a strong convergence, fewer adjustment parameters, and an easy implementation which are shown in some classical benchmark test function [27]. According to the NFL (No Free Lunch) [28] theorem, the grey wolf optimization has the shortcomings of slow convergence and easy local optimum, like the other optimization algorithm [29]. At present, the improvement of grey wolf optimization mainly includes four aspects. First, change the variation rule of parameter α. In the GWO algorithm, a  decreases linearly from two to zero as the number of iterations increases. Long [30] used a logarithmic decay function to dynamically reduce the value of α to enhance the exploitation capability of the algorithm. Luis Rodriguez [31] proposed an improved grey wolf optimization based on fuzzy logic, which had obvious advantages as compared with traditional dynamic algorithms. Second, change the position update equation. Inspired by the PSO algorithm, Long [32] made full use of the location information of the remaining individuals in the wolf pack to update the location, thus enhancing the global survey capability of the algorithm and avoiding local optimum. M. Malik [33] replaced the simple arithmetic average with the weighted sum of the optimal position, finding that the multimodal function optimization effect was better. Third, change the regeneration strategy of the population. Gupta, S. [34] designed a GLF–GWO algorithm in which the alpha wolves update their position through the Levy-flight search mechanism and introduce a greed mechanism to prevent the population from falling into local optimum. Fourth, mix with other algorithms. There are many types of metaheuristic algorithms based on intelligent groups. Various algorithms have different search capabilities. Therefore, mixing with other algorithms is also an improved method. For example, Shaheen [35] proposed to integrate the particle swarm algorithm with the GWO algorithm, which can effectively find the global optimal solution of an optimization problem. Daniel, E. [36] used the cuckoo search algorithm to improve the traditional grey wolf optimization, and applied it to the field of medical image fusion. Compared with the traditional method, there was a significant improvement.

In order to solve the problem that the parameters of the electric pitch system FD of the wind turbine generator set are difficult to optimize, an extreme random forest FD model based on improved grey wolf optimization is proposed. First, the Cosine model is introduced to nonlinearize the linearly changing convergence factor α based on the model. Then, the lens imaging principle in physics is introduced. The mirror wolf of the α wolf is generated and compared with the fitness value of the α wolf, thus avoiding the local optimal solution when the population tends to gather towards the α wolf in the later stage of the algorithm. The grey wolf algorithm optimized based on the above two learning strategies is called the improved grey wolf optimization (Improved Grey Wolf Optimizer, IGWO). IGWO integrates with the extreme random forest electric pitch system FD method of the wind turbine generator set through the fitness function, and defines the accuracy of the binary-classification confusion matrix as the fitness function. IGWO will calculate the minimum value of the fitness function at each iteration until the accuracy requirements are met, or the maximum number of iterations is reached. Then, the optimal parameters are outputted to the Extreme Random Forest model. The data set is divided into a training set and a test set. The training set is used to train the model, and the test set is used to test the model and output the predicted value. The value is compared with the test value to calculate the FPR and FNR of the electric pitch system FD of the wind turbine generator set for Extreme Random Forest optimized by the improved grey wolf optimization.

## 2. Materials and Methods

### 2.1. Grey Wolf Optimization

#### 2.1.1. Algorithms Description

Grey Wolf Optimization is a group metaheuristic algorithm [37] which simulates the predation strategy and hierarchy of wolves in nature, and continuously searches for the optimal value in an iterative method [38]. During the predation process, the distance D between the wolf pack and the prey can be expressed by Equation (1). The wolf pack updates its position according to the distance from the prey, expressed by Equation (2):

(1) $$D=\;\left|CX_{p}\left(t\right)-X\left(t\right)\right|$$

(2) $$X\left(t+1\right)\;=X_{P}\left(t\right)-A\cdot D$$

where $X\left(t\right)$ is the position vector of the wolf, $X_{p}\left(t\right)$ is the position vector of the prey, t is the current iteration steps, and *A* and *C* are coefficient vectors. By adjusting these two vectors, the wolf can reach different positions around the prey. The calculation methods can be represented by Equations (3) and (4):

(3) $$A=2ar_{1}-a$$

(4) $$C=2\cdot r_{2}$$

where a linearly decreases from 2 to 0 during the iterative process, and *r*_1_ and *r*_2_ are random vectors between (0, 1).

Suppose that α wolf, β wolf, and δ wolf have strong observation abilities to the potential escape position of the prey. The whole predation process is dominated by α, β, and δ wolf. The position of α wolf is the best, followed by β and δ wolf. First, determine the distances between α, β, and δ wolf and the prey according to Equations (5)–(7), and then move to the next position according to Equations (8)–(11). Finally, ω wolf updates their position according to the α, β, and δ wolf. On the basis of the above methods, the optimal solution of the optimization objective can be obtained by continuous iteration until the termination condition is satisfied.

(5) $$D_{\alpha }=\;\left|C_{1}\cdot X_{\alpha }-X\right|$$

(6) $$D_{\beta }=\;\left|C_{2}\cdot X_{\beta }-X\right|$$

(7) $$D_{\delta }=\;\left|C_{3}\cdot X_{\delta }-X\right|$$

(8) $$X_{1}=X_{\alpha }-D_{\alpha }A_{1}$$

(9) $$X_{2}=X_{\beta }-D_{\beta }A_{2}$$

(10) $$X_{3}=X_{\delta }-D_{\delta }A_{3}$$

(11) $$X\left(t+1\right)=\frac{X_{1}+X_{2}+X_{3}}{3}$$

where $D_{\alpha },D_{\beta },D_{\delta }$ represent the distances between α, β, and δ wolf and the current candidate wolf, respectively. $X_{\alpha },X_{\beta },X_{\delta }$ represent the position vector of the current population of α wolf, β wolf, and δ wolf, *X* represents the position vector of the grey wolf, and $X\left(t+1\right)$ represents the position vector of the next iteration.

#### 2.1.2. Cosine Model-Based Constriction Factor Change Equation

From the Section 2.1. Equation (3), the contraction factor α in GWO changes linearly. In the GWO algorithm, the value of α determines whether the algorithm is in the development or exploration stage in the iterative process. For performance better intelligent algorithms, they should have strong exploration abilities in the initial stage of iteration and find as many global best points as possible. Therefore, the value of α should be large, and the decreasing trend should be steep. In the latter stage of the iteration, it should have strong exploitation capabilities to ensure the quality and convergence speed of the optimal solution, so the value of α should be small, and the decreasing trend should be gentle. The change trend of the cosine function can be seen from the analysis of the change trend. The dynamic cosine function model of contraction factor is shown in Formula (12):

(12) $$a\left(t\right)=1+cos\left(t/Maxiter\cdot \pi \right)$$

where t is the current number of iterations, and Maxiter is the maximum number of iterations.

#### 2.1.3. Grey Wolf Optimization based on Lens Imaging Learning Strategy

**Figure 1:**
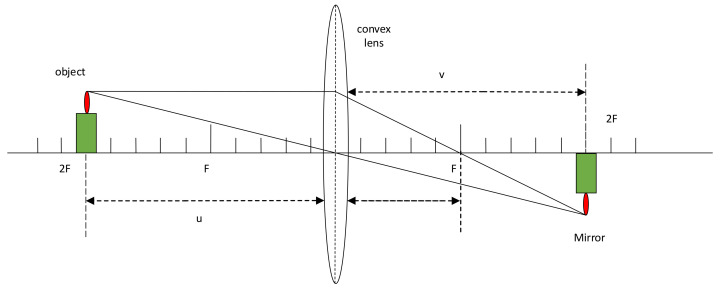
Schematic diagram of lens imaging.

The principle of lens imaging is that an object is refracted by a convex lens, and an image that is opposite to the original object is generated at the other end of the convex lens. Its position and size are determined by the object distance and image distance of the convex lens, as shown in Figure 1.

(13) $$\frac{1}{u}+\frac{1}{v}=\frac{1}{f}$$

where u is the object distance, v is the image distance, and f is the focal length of the lens.

**Definition** **1.** *Mirror point: the sample of M-dimensional space is*$X=\left(x_{1},x_{2},\cdots ,x_{M}\right)$*,*$x_{i}\in \;\left[A_{i},B_{i}\right]$*,*i=1,2,⋯,M*. According to the principle of lens imaging, it can generate the mirror point*$X^{\prime }=\left(x_{1}^{\prime },x_{2}^{\prime }\cdots ,x_{M}^{\prime }\right)$*and*$x_{i}^{\prime }=A_{i}+B_{i}-x_{i}$.

**Definition** **2.** *Base point: The Euclidean distance between the sample point and the mirror point and a certain point O in the M-dimensional space is* d *and* $d^{\prime }$ *. If* $d/d^{\prime }$ *is an integer, the point O is called the base point.*

**Figure 2:**
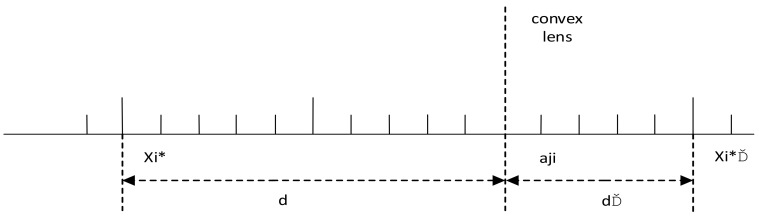
One dimensional inverse learning strategy diagram based on lens imaging principle.

Suppose that the optimal solution is $X^{*}=\left(x_{1}^{*},x_{2}^{*}\cdots ,x_{M}^{*}\right)$. According to Definitions 1 and 2, it can be found that the mirror image point of $X^{*}$ is $X^{*\prime }=\left(x_{1}^{*\prime },x_{2}^{*\prime }\cdots ,x_{M}^{*\prime }\right)$. Suppose that the base point $o_{j}$ is $\left(a_{j1},a_{j2},\cdots ,a_{jM}\right)$. In this case, the mirror point $X^{*\prime }$ can be calculated according to Equation (12). Figure 2 shows the reverse learning strategy based on lens imaging in one-dimensional space.

During the iterative process of the grey wolf optimization, the population of wolves tends to converge toward the head wolf and fall into the local optimal solution. The reverse group is generated through the lens imaging learning strategy, which increases the diversity of the group and avoids the local optimum solution, thus improving the local exploitation ability in the later stage of the algorithm.

### 2.2. Random Forest

Random Forest (RF) is an ensemble model composed of multiple decision trees $\left\{h\left(x,\theta_{k}\right)|k=1,2,3\ldots n_{tree}\right\}$, where $\theta_{k}$ is an independent and identically distributed random vector. The basic ideas and steps of the RF model are as follows.

First, RF randomly selects *K* times from the sample set $D\left(X,Y\right)$ through sampling with replacement, and obtains *K* sample subsets with the same dimensions as the sample set $\left[D_{1},D_{2},\cdots ,D_{k}\right]$.

Then, RF uses the CART decision tree as the weak learner. For each sample in the *N* × *M* dimensional sample set $D\left(X,Y\right)$, there are *M* attributes, which are selected based on the Gini coefficient. The criterion for the selection of the Gini coefficient is that each child node achieves the highest purity. In this case, the Gini coefficient is the smallest, the purity is the highest, and the uncertainty is the smallest. The calculation equation of Gini coefficient is as follows:

(14) $$Gini\left(D\right)=\overset{\left|y\right|}{\underset{k=1}{\sum }}\;\underset{k^{\prime }\neq k}{\sum }p_{k}p_{k^{\prime }}=1-\overset{\left|y\right|}{\underset{k=1}{\sum }}P_{k}^{2}$$

where $p_{k}$ indicates that the proportion of the *k-th* sample in the current sample set $D\left(X,Y\right)$. The Gini coefficient of the attribute α is defined as:

(15) $$G_{ini\_index}\left(D,a\right)=\overset{V}{\underset{\nu =1}{\sum }}\frac{\left|D^{v}\right|}{\left|D\right|}G_{ini}\left(D^{\nu }\right)$$

Therefore, in the candidate attribute set A, the optimal partition attribute minimizes the Gini index after partitioning, namely $a^{*}=arg\min G_{ini\_index}\left(D,a\right)$.

Finally, repeat the above steps to build multiple decision trees to form a random forest. Input the prediction data into the constructed RF model where multiple decision trees make decisions at the same time, and make category decisions based on the principle that the minority obeys the majority.

### 2.3. Extreme Random Forest

Extreme random trees (ERT) is a derivative algorithm of RF. It is a machine learning algorithm proposed by Pierre geurts and other scholars after a lot of experimental research in 2006. Extreme random forest (ERF) is also a classifier integrated by multiple decision trees, but compared with RF, ERF is better in classification accuracy and training time, which is mainly due to its two differences from RF:

(1) The training set of decision tree is obtained in different ways. RF adopts bagging model, which has put back randomly selected equal dimensional training set, and its training set is random, but there may be duplicate samples in the training set, which can not ensure that all samples are fully utilized, and there may be similarity between two arbitrary training sets, resulting in that the trained classifiers can not play their respective functions. The training set of ERF does not use random sampling, but uses all the original training sets, that is, each decision tree applies the same all training samples, which ensures the utilization of training samples and reduces the final prediction deviation to a certain extent.
(2) Characteristics are divided in different ways. The decision tree of RF will select an optimal eigenvalue for division based on the principles of information gain, Gini coefficient and mean square deviation. For example, in candidate attribute set A, select the attribute that minimizes the Gini index after division as the optimal division attribute, $argminG_{ini\_index}\left(D,a\right)$. ERF has strong randomness for the acquisition of splitting features and segmentation values. It randomly selects an eigenvalue for division, so that each decision tree presents structural differences

The algorithm principle of ERT is the same as RF except that the acquisition of training set and feature division are different. It scores by integrating multiple decision trees and votes according to the average value of the predicted value of each decision tree. The process is as follows:

(1) Sample selection. Each decision tree is trained with the original data set.
(2) Select the partition feature. ERT randomly selects an eigenvalue to divide the decision tree. For N*M dimensional sample set *D (x, y)*, given sample *x_i_* use *m* dimensional eigenvector *f_i_* represents the characteristics of the sample. Then, a partition value $a_{c}$ is randomly selected between at the maximum value of the variable $a_{max}^{K}$ and minimum variable of $a_{min}^{K}$ partition. If the value of variable *k* less than the split value $a_{c}$ sample $\left(a<a_{c}\right)$, then put them in the left leaf node, and if the value of variable k is greater than or equal to the split value $a_{c}$ *sample* $\left(a\geq a_{c}\right)$, then put them in the right leaf node.
(3) Build the decision tree. The formation of the decision tree is divided and split according to the division rules in step (2) until it can no longer be split.
(4) Extreme random tree prediction. Repeat steps (1), (2) and (3) to establish a large number of decision trees and gradually form a forest until the number of iterations is met. Input the prediction data into the constructed forest, calculate the output results of each decision tree for classification or regression, and get the final classification or regression prediction results.

The selection of feature *K* and threshold $a_{c}$ in N* M dimensional sample set *D* (*x*, *y*) according to Formula (16). Calculating the score measurement of each feature, and selecting the highest score as the splitting feature and splitting threshold of the leaf node.

(16) $$Score_{c}\left(k,S\right)=\frac{2I_{c}^{k}\left(S\right)}{H_{k}\left(S\right)+H_{c}\left(S\right)}$$

where $I_{c}^{k}\left(S\right)$ indicates that node *S* was splitted based on characteristic *K* and threshold $a_{c}^{k}$, the two subsets have mutual information about categories. $H_{k}\left(S\right)$ represents the splitting entropy of characteristic *K*. $H_{c}\left(S\right)$ represents the information entropy of node S about the category. Compared with Gini index and information gain, this index introduces $H_{c}\left(S\right)$ and $H_{k}\left(S\right)$ symmetry reduces the influence of class distribution on node splitting. Formula (15) represents the fractional measure of feature *K* on leaf node *S*. The final probability of each sample is the probability average of all trees, which is defined as follows:

(17) $$P_{\left(c|f_{i}\right)}=\frac{1}{M}\overset{M}{\underset{t=1}{\sum }}P_{t}\left(c|f_{i}\right)$$

(18) $$\hat{c}={\mathrm{argmax}}_{c}\;P\left(c|f_{i}\right)$$

where M is the total number of trees, $f_{i}$ represents eigenvector of sample x_i_, P_t_ is expressed conditional probability that the sample belongs to category C under the condition of in vector $f_{i}$. Formula (17) defines the classification probability of samples in the decision tree. Finally, on the extreme random forest, Formula (18) uses the voting principle to determine the category of the sample.

### 2.4. Wind Turbines Pitch System FD of Random Forest based on Improved Grey Wolf Optimization

**Figure 3:**
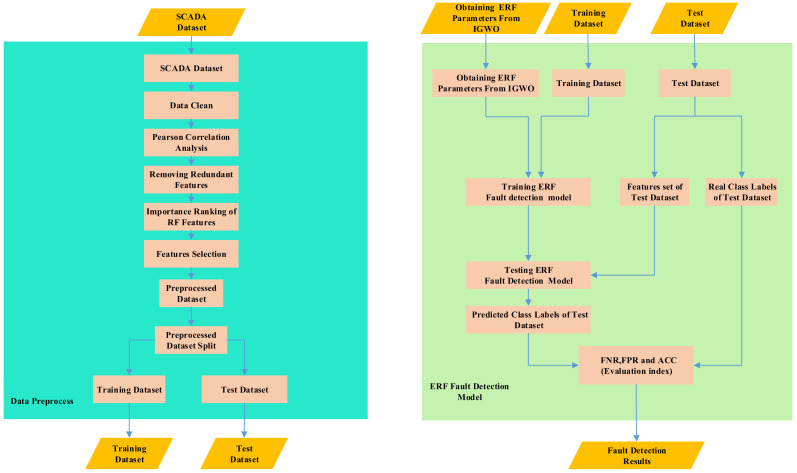
The flow chart of Data Preprocess and ERF Fault Detection Model.

**Figure 4:**
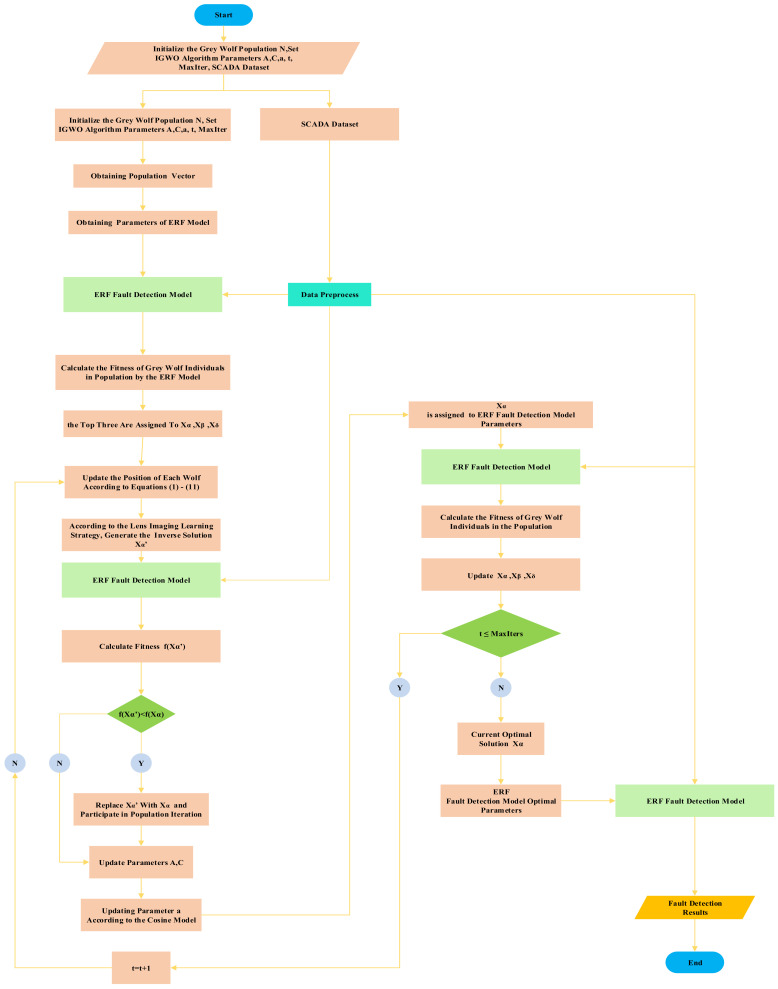
FD flow chart of wind turbine electric pitch system based on IGWO-ERF.

Because the SCADA system records various historical data of the wind turbine during operation—including normal data and various fault data over a period of time, before the model is trained—operations such as preprocessing and feature selection should be performed on the extracted data to remove redundant variables and to reduce the complexity of model training. The steps in the solid box on the left in Figure 3 represent the preprocessing process. First, data cleaning, then Pearson correlation analysis is used to eliminate redundant variables, then Random Forest is used to sort the importance, and the features of the first *N* are selected to construct the data set. The steps in the light green solid box on the right in Figure 3 represents the extreme random forest fault detection model. The input of the model includes the parameters optimized by IGWO, training set and test set, and the output includes the evaluation index of FNR, FPR and fitness function ACC. Figure 4 is the fault diagnosis flow chart of wind turbine pitch system. Firstly, the steps of population initialization, parameter initialization and data preprocessing are used as the input of ERF fault detection model, and then the fitness function ACC is output to calculate the fitness function value of each individual of gray wolf population, and assign the top three to α, β, and ω wolf. Then update the position of grey wolf individual according to Formulas (1)–(11). Then, according to the lens imaging reverse learning strategy, the learning strategies were reversed to generate α’ wolf and to calculate its fitness value as compared with α wolf’s fitness value. If the α’ wolf’s fitness value was lower than the α wolf, α’ Wolf was substituted for α wolf and participated in the population iteration process, *A, C* were updated by the original grey wolf algorithm, and *a* was updated according to the Cosine model. After updating the parameters, the X_α_ and the preprocessed data are substituted into the ERF fault detection model, calculating the fitness of grey wolf population individuals, updating the positions of α, β and δ wolf, and the above steps are repeated until the maximum iteration requirements are met.

#### 2.4.1. Data Preprocessing

The SCADA system records the operating status of each system of the wind turbine generator set. The records mainly include wind speed, wind direction, temperature of each component, power, rotor speed, etc. Since the wind turbine operating environment is relatively harsh, the data recorded by the SCADA system has problems such as null values and abnormal values. The electric pitch system has many internal parameters: there are differences in the nature, dimension, and unit of each parameter. Therefore, after the initial cleaning of the original data extracted by the SCADA system, the Z-score method is needed to scale the data proportionally, and the data of the same parameter is processed into the interval (−1, 1) with the mean u=0 and the variance σ=1. The Equation (19) is the mathematical expression of the Z-score, and the variance calculation is shown in Equation (20).

(19) $$z=\frac{x-u}{\sigma }$$

(20) $$\sigma =\sqrt{\frac{1}{N}\overset{N}{\underset{i=1}{\sum }}{\left(x_{i}-\mu \right)}^{2}}$$

During the optimization of the Extreme Random Forest parameters using the improved grey wolf optimization, the above two parameters (Table 1) are set into a two-dimensional vector X(t+1) (n_estimators, min_samples_leaf). The fitness of each individual is calculated during each iteration of the algorithm. If the fitness after iteration is better than the current stage, replace it. Otherwise, discard the current state vector and proceed to the next iteration until the maximum number of iterations is met or the accuracy requirement is met. The pseudo code of the optimized Extreme Random Forest parameters based on improved grey wolf optimization is shown in Algorithm 1.
**Algorithm 1.** IGWO algorithm optimizes Extreme Random Forest**Input:***N, Maxiters, k, t* **Output**:
$X_{\alpha }$
1. $X_{\alpha },\;X_{\beta },\;X_{\delta },\;X_{w}\leftarrow X_{0}$ 2. **repeat** 3. Surround, The parameters *A* and *C* are calculated according to Formulas (3) and  (4) 4. Hunting, $X_{t+1}$ is calculated according to Formulas (3) and (4), $\;X_{t+1}\left(n\_estimators,min\_samples\_leaf\right)\leftarrow X_{t}\left(X_{\alpha },\;X_{\beta },\;X_{\delta },X_{w},A,C\right)$ 5. $\mathrm{RF}\leftarrow X_{t+1},\;$ training set 6. $f\left(X_{\alpha }\right)\leftarrow ACC\leftarrow RF\leftarrow$ test set 7. Track, Assign according to the greed criterion,  $BestX_{\alpha },\;BestX_{\beta },BestX_{\delta }\leftarrow X_{\alpha },\;X_{\beta },\;X_{\delta },X_{P}$
8. $X_{\alpha }^{\text{'}}\leftarrow BestX_{\alpha }$ 9. $\mathrm{ERF}\leftarrow (X_{\alpha }^{\prime },\mathrm{test}\;\mathrm{set}$ training set) 10. $f\left(X_{\alpha }^{\prime }\right)\leftarrow ACC\leftarrow RF\leftarrow \mathrm{test}\;\mathrm{set}$ 11. **If** $f\left(X_{\alpha }^{\prime }\right)$ < $f\left(X_{\alpha }\right)$ **then** 12. $BestX_{\alpha }\leftarrow X_{\alpha }^{\prime }$ 13. **end if** 14. **until**
*Maxiter* 15.  print $X_{\alpha }$
16. $ERF\left(n\_estimators,min\_samples\_leaf\right)\leftarrow BestX_{\alpha }$ 17. $ERF\left(n\_estimators,min\_samples\_leaf\right)\;\leftarrow$ training set 18. $Y\_pred\leftarrow RF\left(n\_estimators,min\_samples\_leaf\right)\leftarrow$ test set 19. FPR,FNR←confusion_matrix (Y_pred, Y_test)

#### 2.4.2. Fault Detection Performance Evaluation Index

To verify the effectiveness of the optimized Extreme Random Forest model based on the improved grey wolf, the optimized Extreme Random Forest based on the Particle Swarm Optimization (PSO) [39], Sine and Cosine Optimization Algorithm (SCA) [40], Harris Hawks Optimization (HHO) [41], and Improved Grey Wolf Optimization (IGWO) are applied to the electric pitch system FD model of wind turbines for comparison. The research objects are the high temperature fault of the pitch super capacitor, the high temperature fault of the pitch shaft box, and the main power supply fault of the pitch. For the binary-classification problem of the electric pitch system FD of wind turbines, a confusion matrix is introduced, as shown in Table 2. The false positive rate and the false negative rate of the model are used as evaluation indexes. Equation (21) represents the false positive rate, and Equation (22) indicates the false negative rate.

(21) $$FPR=\frac{FP}{TN+FP}$$

(22) $$FNR=\frac{FN}{TP+FN}$$

### 2.5. Experimental Analysis

#### 2.5.1. Data Description

The experiment selects the actual operating data of a wind farm in Inner Mongolia from January to June 2021. There are 33 sets of 1.5 MW double-fed wind turbines in the wind farm, and the data sampling interval is 60 s. Partial operation data is shown in Table 3. Because the working conditions of the wind turbine generator set are easily affected by the surrounding environment—especially the uncertainty of wind speed changes [42]—the electric pitch system should automatically adjust the angle between the blades and the wind direction in accordance with the wind speed, so that the generator has a rated output power and to ensure the safe operation of the entire wind turbine generator set.

#### 2.5.2. Sample Feature Selection

First, the collected data is performed with data processing to delete the data whose state quantity is “0” and unchanged. The Z-score [43] is used for normalization processing. The Pearson correlation coefficient is used to conduct the correlation analysis with Converter_power. The results are shown in Table 4. Because the electric pitch system controls the speed of the wind wheel by controlling the angle of the blades, thus controlling the output power of the wind turbine, the output power of the electric pitch system is selected as the main electric of the Pearson correlation analysis.

**Figure 5:**
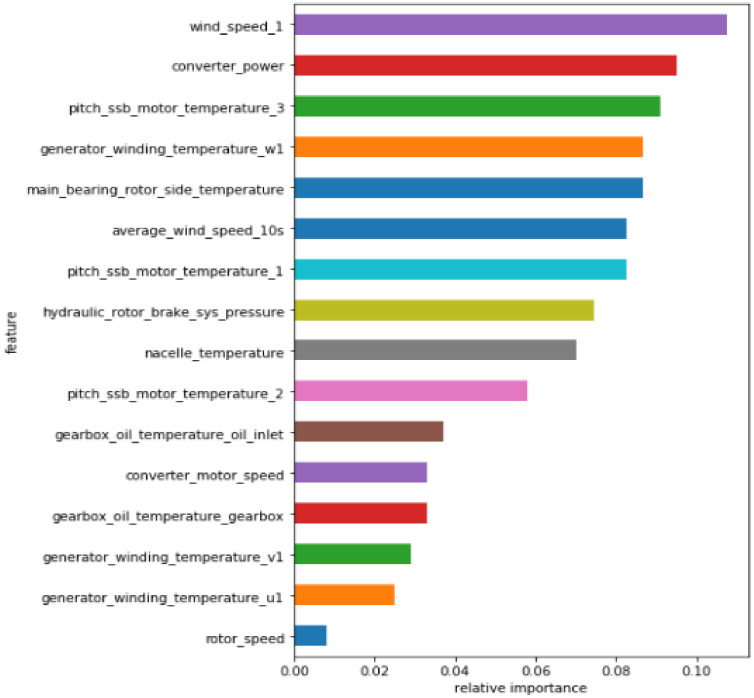
Random forest feature importance ranking diagram.

The analysis of Table 4 shows that the correlation between different characteristic variables and Converter_power is different. The characteristic variables with an absolute value of a Pearson correlation coefficient greater than 0.6 (such as the bolded part in Table 4) are retained, and redundant variables with a lower correlation are eliminated. The RF is used to conduct the importance ranking of the data set after the Pearson screening. The top N = 8 features are selected as the main influencing factors of the electric pitch system failure. The feature importance ranking of RF is shown in Figure 5.

## 3. Results

**Figure 6:**
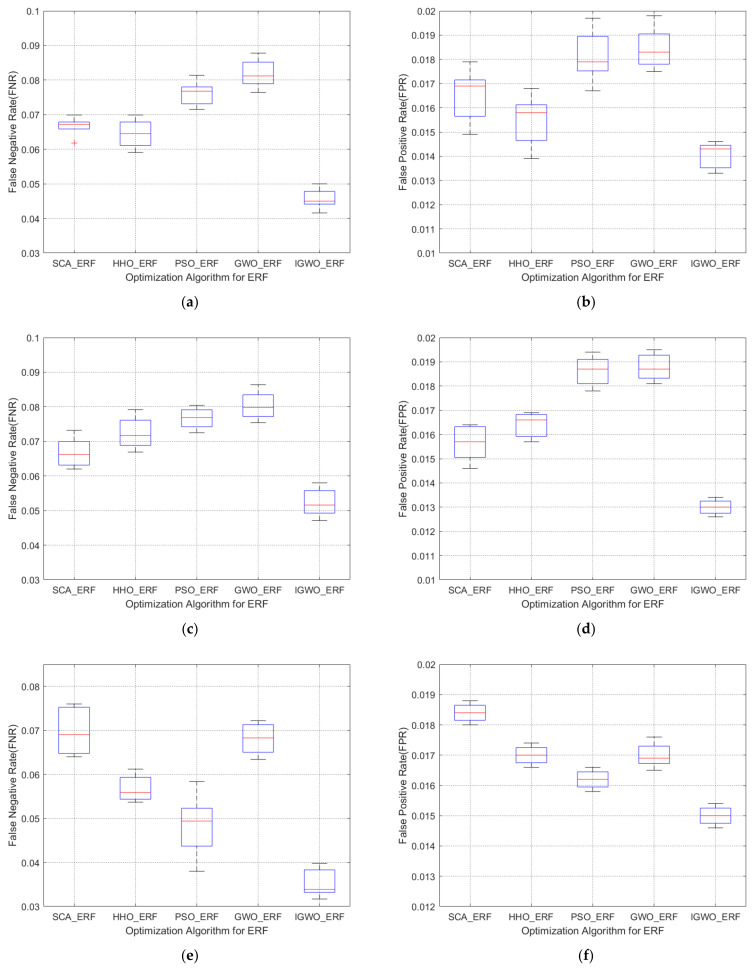
(**a**) The FNR box diagram of the supercapacitor fault; (**b**) the FPR box diagram of the supercapacitor fault; (**c**) the FNR box diagram of electric pitch axle box fault; (**d**) the FPR box diagram of electric pitch axle box fault; (**e**) the FNR box diagram of electric pitch main power; and (**f**) the FPR box diagram of electric pitch main power.

To compare IGWO with SCA, HHO, and PSO, these algorithms are substituted into the wind turbine generator set electric pitch system FD. The initial populations of the four optimization algorithms are all set to 30, the maximum number of iterations is set to 500, and the experiments are performed 10 times. The FPR and the FNR of the overall experiment are used to draw a box plot. The high temperature fault of the pitch supercapacitor are shown in Figure 6a,b. The high temperature failures of the pitch shaft box are shown in Figure 6c,d. The main power supply failures of the pitch are shown in Figure 6e,f.

From the box diagrams of FPR and FNR of the three types of faults, it can be known that the FD model of the wind turbine pitch system with four optimization algorithms optimized with Extreme Random Forest parameters has lower false positive rates and false negative rates and, compared with GWO, IGWO has a better optimization effect. The false negative rates of the three types of faults are all smaller than 10%, and the false positive rates are all smaller than 2.5%. For the high temperature fault of the pitch supercapacitor, the false positive rates of SCA, HHO, and PSO optimization algorithms are within 6–8%, and the false negative rates are within 1.5–2%. The false positive rate of IGWO is within 4–5% and the false positive rate is within 1.3–1.5%. The experimental results of the high temperature fault of the rotor axle box show that the Extreme Random Forest effect of PSO optimization is not obvious, the optimization effect is very weak, and the overall optimization performance of IGWO is good. The results of the FD of the main power supply of the pitch change show that, compared with SCA, HHO, and PSO optimization algorithms, the false negative rate of IGWO is reduced by 1–5%, and the false positive rate is reduced by 0.2–0.4%. Based on the above analysis, it is evident that IGWO has a lower false positive rate and false negative rate as compared with SCA, HHO, and PSO, indicating that the wind turbine generator set electric pitch system FD model of Extreme Random Forest, whose parameters are optimized by the IGWO, has a better performance.

## 4. Conclusions

It is difficult to optimize the parameters of a double-fed wind turbine generator set electric pitch system fault model in Extreme Random Forest detection. To solve this problem, an improved double-fed wind turbine generator set electric pitch system FD model that optimizes the parameters of the Extreme Random Forest is proposed. The main contributions of this paper are threefold. First, it introduces the Cosine model. Based on the change of the convergence factor α in the algorithm iteration process, the Cosine model is integrated with the GWO to balance the exploitation and exploration capabilities. Second, it introduces the lens imaging reverse learning strategy. The optimal solution of α wolf will generate its mirror wolf based on the lens imaging learning strategy. This strategy compares the fitness values to avoid the algorithm falling into a local optimal solution at the later stage of the iteration. Third, the above two learning strategies are combined to propose an improved grey wolf optimization, which is combined with Extreme Random Forest and applied to the wind turbine generator set electric pitch system FD model. The improved grey wolf optimization, sine cosine optimization algorithms, Harris hawks optimization algorithm, and particle swarm optimization algorithm are combined with Extreme Random Forest, respectively. The accuracy of the confusion matrix is used as the fitness function, and the false positive rate and false negative rate are used as the evaluation index. The experimental results show that the improved grey wolf optimization has a lower false positive rate and a lower false negative rate, indicating that the wind turbine generator set electric pitch system FD method of Extreme Random Forest optimized by the improved grey wolf optimization has good performance. The reason why the IGWO–RF model has good performance in the fault detection of the wind turbine electric pitch system is that the RF model has a reasonable logical design, and the RF without any optimization algorithm can achieve good results in the field of fault detection. After the IGWO–RF model optimizes Extreme Random Forest, the model performance has been improved, but the qualitative change of magnitude has not necessarily been reached. The original data in the SCADA system is subject to a high degree of processing to use it, and the data preprocessing method is more, which cannot unite a measure. In view of the above problem, we still need to spend more time studying and formulating conforms to the engineering practice of the electric pitch system fault detection model. There is still a long way to go.

## Figures and Tables

**Table 1:** RF parameter: n_estimators and min_sample_leaf.

Parameter	Meaning	Value Range
n_estimators	The number of weak classifiers parameter, which is used to adjust the number of trees	(4200)
min_sample_leaf	Minimum leaf node sample number, which is used to adjust the minimum sample number of leaf nodes of the base classifier	(1300)

**Table 2:** Confusion matrix of dichotomies problem.

The Actual Category	The Predict Category	
	Normal	Fault
Normal	TP	FN
Fault	FP	TN

**Table 3:** Partial operation data of fan No. A16 on 1 February 2021.

State Parameter	Time									
	00:00:00	00:01:00	00:02:00	…	08:27:00	08:28:00	08:29:00	…	08:48:00	08:49:00
rotor_speed/(r∙m^−1^)	17.383	17.338	17.586	…	16.88	17.492	17.538	…	17.567	17.246
converter_motor_speed/(r∙m^−1^)	1746.6	1751	1744.8	…	1696.1	1757.6	1762.2	…	1765.1	1732.9
…	…	…	…	…	…	…	…	…	…	…
pitch_ssb_motor_current_2/A	15.686	4.412	9.314	…	19.1176	3.9216	4.4118	…	2.9412	13.725

**Table 4:** Pearson correlation coefficient between each parameter and Converter_power.

State Parameter	Pearson Correlation Coefficient	State Parameter	Pearson Correlation Coefficient
rotor_speed/(r∙m^−1^)	**0.83827**	generator_winding_temperature_u1/°C	**0.67398**
converter_motor_speed/(r∙m^−1^)	**0.83846**	pitch_ssb_motor_current_1/A	0.47505
…	…	…	…
nacelle_temperature/a	**−0.75862**	hydraulic_main_sys_pressure/N	−0.35687

## Data Availability

The data that support the findings of this study are available from the corresponding author, upon reasonable request.
